# Determining the Stationarity Distance via a Reversible Stochastic Process

**DOI:** 10.1371/journal.pone.0164110

**Published:** 2016-10-20

**Authors:** Marios Poulos

**Affiliations:** Laboratory of Information Technologies, Faculty of Information Science and Informatics, Ionian University, Corfu, Greece; Tianjin University, CHINA

## Abstract

The problem of controlling stationarity involves an important aspect of forecasting, in which a time series is analyzed in terms of levels or differences. In the literature, non-parametric stationary tests, such as the Kwiatkowski-Phillips-Schmidt-Shin (KPSS) tests, have been shown to be very important; however, they are affected by problems with the reliability of lag and sample size selection. To date, no theoretical criterion has been proposed for the lag-length selection for tests of the null hypothesis of stationarity. Their use should be avoided, even for the purpose of so-called ‘confirmation’. The aim of this study is to introduce a new method that measures the distance by obtaining each numerical series from its own time-reversed series. This distance is based on a novel stationary ergodic process, in which the stationary series has reversible symmetric features, and is calculated using the Dynamic Time-warping (DTW) algorithm in a self-correlation procedure. Furthermore, to establish a stronger statistical foundation for this method, the F-test is used as a statistical control and is a suggestion for future statistical research on resolving the problem of a sample of limited size being introduced. Finally, as described in the theoretical and experimental documentation, this distance indicates the degree of non-stationarity of the times series.

## Introduction

A time series is a sequence of data points collected over time, and the analysis of time series is a very important issue in economic and engineering forecasting. In the engineering literature, state-space methods have been developed for sequential data analysis. Several researchers have attempted to bridge the gap between engineering methods and statistics [1, 2]. Recently, two advanced research areas have been combined using this approach: the methods of nonlinear time-series analysis and the theory of complex networks. Multivariate time-series analysis is used to model and explain the interactions and co-movements among a group of time-series variables. Specifically, Gao et al. [3–6] recently developed some important multivariate time series analysis methods, especially complex network-based methods, to uncover complicated flow mechanism underlying industrial multiphase flow system. Time-frequency (TF) analysis is considered to be one of the most significant active fields with respect to this issue. Because such data are essentially non-stationary and correlated and often have periodic patterns, multivariate time series are difficult to analyze and forecast [1].

In time-series clustering, time series data are partitioned into groups based on similarity or distance so that time series in the same cluster are similar. For time-series clustering, the first step is identifying an appropriate distance/similarity metric. Then, in the second step, existing clustering techniques, such as k-means, hierarchical and density-based clustering or subspace clustering, are used to find clustering structures. Furthermore, with modern methods, such as multivariate multi-scale complex network (MMCN) analysis [6], mapping a multivariate time series into a MMCN can be achieved. The goal of this study is to investigate the inherent properties of multivariate time series from the perspective of complex-network and multi-scale analysis. One significant property of complex network analysis is stationarity.

The stationarity property of a time series can be used to obtain significant sample statistics, such as variances, means, and correlations with other variables. However, such statistics are valuable as descriptors of forthcoming behavior only if the series is stationary. For instance, if the series reliably increases over time, the sample mean and variance will increase with the sample size but will always undervalue the mean and variance in forthcoming periods.

A stationary process is a process whose statistical properties (mean and standard deviation) do not vary according to the place or the time (at which the function is calculated) [7]. There are two main types of methods for examining stationarity: the parametric method and the nonparametric method [8]. Parametric approaches are generally used by researchers working in the time domain, such as economists, who make certain assumptions about the nature of their data. The bases of these approaches are unit-root tests (beginning with the classic Dickey-Fuller [DF] test, with numerous modifications, and Perron-type tests), which have as a null hypothesis the existence of a unit root in the series. These approaches use parametric auto-regression to approximate the autoregressive-moving-average (ARMA) structure of the errors in the test regression. The alternative to stationarity is a combined hypothesis. There are many types of Kwiatkowski-Phillips-Schmidt-Shin (KPSS) tests, such as the popular ones used for testing integration, which take null stationarity as a simple hypothesis [9]. The difference between these tests is that the KPSS test uses a nonparametric method to correct a series for serial correlation, whereas the DF and Perron tests use parametric corrections [9].

Given a choice between parametric and nonparametric tests, scientists choose the latter because the assumptions made in KPSS tests are more general, which makes them applicable to a wider class of processes. However, in both cases, problems remain unsolved. One application of KPSS tests is the adaptive detection of bandwidth through an observation process [10]. Additionally, in nonparametric methods, choosing the appropriate order (lag) for describing the time series is a critical step. This choice requires lag selection that satisfies stationary control, such as a KPSS test. Furthermore, the order (lag) selection is extremely difficult when the experiment focuses on only a few sample data; in this case, the default order is zero (0).

Additionally, an increase in sample size does not help reduce the occurrence of rejections; in fact, it increases the rejection rate. This finding runs counter to the large-sample theory on which stationarity tests typically rely: Asymptotic approximations yield higher accuracies as the sample size increases. The existence of such a size problem immediately calls into question the credibility of examining empirical evidence with stationarity tests, such as KPSS. Indeed, it is well understood that many observed time series in empirical macroeconomics and international finance exhibit robust persistence and, thus, fall into the problematic parameter zone [11].

Furthermore, the results of experiments conducted on Indian macroeconomic variables revealed that the aforementioned tests, including KPSS tests, are prohibitively sensitive to the choice of lag length [6]. Since, as of now, no theoretical criterion exists for the lag-length selection for tests in which the null hypothesis is stationarity, their use should be avoided, even for so-called ‘confirmation’ [12].

In conclusion, parametric stationary tests, such as KPSS tests, are very important but suffer from problems with reliability in the lag and sample size selection that limit their applicability.

The aim of this study is to introduce a new method that measures the distance in terms of the measurement error between mirror time series. This error corresponds to the theoretical distance that must be investigated to determine whether a time series is stationary. Using this approach eliminates the aforementioned problems with lag and sample size selection. Furthermore, the distance calculated via this method could be used as a specific property in a multi-scale complex network.

This distance is based on a stationary ergodic process, which relies on the following three (3) considerations:

1. Any reversible process is stationary, and any time reversal of a reversible process is also stationary [13–15].
2. If {*X*_*n*_,*n* ∈ *R*} is stationary, then the time-reversed process $\left\{{\tilde{X}}_{n},n\in R\right\}$ is also stationary.
3. The possible metric deviation (distance) between the unpredictable series $Y_{n}\&{\tilde{Y}}_{n}$ can be used as a measure of the degree of irreversible progress; this is implemented using the Dynamic Time-warping (DTW) technique. DTW is adopted because it is considered to be the better of the two time-series methods [16] because its metric is superior to the classic Euclidean distance metric. This metric, which corresponds to the degree of irreversibility, is called the “Stationarity Distance”, and it is a measure of the non-stationary characteristics of a time series and is used in the above-described process.

To corroborate these assertions, a series of statistical procedures is applied using the proposed method to analyze a specific data set. Additionally, a KPSS test (with a null hypothesis of stationarity) is also applied to the same data [17], and the reliability of the proposed method is evaluated on suitable (with stationary and non-stationary properties) time-series data. For further verification, the selected (non-)stationary data segments are visually inspected by plotting. More details on this procedure are presented in the Experimental Procedure section.

Finally, this study can be broadly divided into four sections. In the first section, Methodology, the definitions of the reversible stochastic process (RSP) and KPSS methods are given. In the second section, Experimental Procedure, the experimental methods and the results obtained with both methods are presented. In the third section, the statistical evaluation of the RSP method is analyzed. Finally, in the fourth section, the conclusions and plans for future research on the RSP method are described.

## Formulation

### The RSP Method

This method is based on the following formulation:

A discrete time-stationary process {*X*_*N*_,*i* = 1,…,*N*} is time reversible if, for every positive integer N [14],

$$\left(X_{1},X_{2},\ldots X_{N}\right)=\left(X_{N},X_{N-1},\ldots X_{1}\right),$$ (1)

and a discrete time series $\left\{{\vec{X}}_{N},i=0,1,\ldots ,N\right\}$ produces a corresponding mirror time series

$$\left\{Y_{N}={\overset{\leftarrow }{X}}_{N},i=0,1,\ldots ,N\right\},$$ (2)

$$\begin{array}{l} \overset{N}{\underset{i=1}{X}}=\left\{x_{1},x_{2,}\ldots ,x_{N-1},x_{N}\right\}\\ \text{where}\\ \overset{N}{\underset{i=1}{Y}}=\left\{y_{1},y_{2,}\ldots ,y_{N-1},y_{N}\right\}=\left\{x_{N},x_{N-1},\ldots ,x_{2},x_{1}\right\} \end{array}$$ (3)

Thus, given Eq 2, the proposed algorithm is based on the following:

$${\vec{X}}_{N}={\overset{\leftarrow }{X}}_{N}-error.$$ (4)

If the error is zero (*error* = 0), then the time series ${\vec{X}}_{N}$ consists of a stationary process, as defined in Eq 1, where the estimated error is based on the dissimilarity measure between the discrete time series ${\vec{X}}_{N}$ and the reversible time series ${\overset{\leftarrow }{X}}_{N}$. The physical meaning of this distance is the process’s degree of stationarity.

### Error calculation using DTW

Numerous time series dissimilarity measures have been proposed [18]. However, the most common measures, which were proposed long ago, remain the most competitive ones. The most-used metric distance function is the Euclidean distance, which obeys the metric properties: *triangle inequality*, *non-negativity*, *identity*, and *symmetry*. This function remains amazingly competitive [19] with other, more complicated metrics, particularly for large data sets [20]. Euclidean distance and its variants have several drawbacks that make their use inappropriate in certain applications, such as sensitivity to noise, shifting and scaling [19,21]

DTW lends robustness to the similarity computation. Using this method, time series of different lengths can be compared because DTW replaces the one-to-one point comparison, used in Euclidean distance, with a many-to-one (and vice versa) comparison [19]. Thus, one of the main conclusions of comparison studies [22,23] is that, despite newly proposed methods, the Euclidean and DTW [20,24] dissimilarity measures remain two of the most robust, simple, generic, and capable measures. Additionally, to measure the shape similarity in the sign data set, alignment-based distances (such as DTW) are more suitable; in contrast, the Euclidean distance is not robust to distortions in time and other noise [25]. Because the DTW algorithm is more efficient than the Euclidean distance with respect to noise sensitivity [20,24,25], the DTW is selected for use in the error calculation based on Eq 3 to obtain more reliable measurements than would be possible using other, similar techniques.

Assume that the local dissimilarity of function {*f*} is defined between any pair of elements *x*_*i*_ ∧ *y*_*i*_ under the constraint

$$d\left(i,j\right)=f\left(x_{i},y_{i}\right)\geq 0.$$ (5)

Then, if a given path is the lowest-cost path between two series, the corresponding technique (DTW) [26] lays the warping curve *φ*(*k*), ∀ *k* = 1,2,…,*T*:

$$\begin{array}{l} \varphi \left(k\right)=\left(\varphi_{\chi }\left(k\right),\varphi_{y}\left(k\right)\right)\;with\\ \varphi_{\chi }\left(k\right)\overset{}{}\;\wedge \;\varphi_{y}\left(k\right)\in \left\{1,2,\ldots ,N\right\} \end{array}.$$ (6)

The warping functions *φ*_*x*_ ∧ *φ*_*y*_ remap the time indices of *X* ∧ *Y* correspondingly. Given *φ*, the average accumulated distortion between the warped time series *X* ∧ *Y* can be calculated [26] as follows.

$$d_{\varphi }\left(X,Y\right)=\sum_{\kappa =1}^{T}\frac{d\left(\varphi_{x}\left(k\right),\varphi_{x}\left(k\right)\right)m_{\varphi }\left(\kappa \right)}{M_{\varphi }},$$ (7)

where *m*_*φ*_(*k*) is a per-step weighting coefficient, and *M*_*φ*_ is the corresponding normalization constant, which confirms that the accumulated distortions are comparable along different paths. To ensure reasonable warps, constraints on *φ* are usually imposed. The idea underlying DTW is finding the optimal alignment *φ* such that

$$D\left(X_{N},Y_{N}\right)=\underset{\varphi }{\min}\left\{d_{\varphi }\left(X_{N},Y_{N}\right)\right\}.$$ (8)

Therefore, one chooses the distortion of the time axes of *X* ∧ *Y* that brings the two time series as near to each other as possible.

### Procedure formulation

The measure of the degree of non-stationarity is calculated according to the scaling of $\underset{\varphi }{\min}\left\{d_{\varphi }\left(X_{N},Y_{N}\right)\right\}>0$, keeping in mind that a time series {*X*_*N*_} is stationary when $\underset{\varphi }{\min}\left\{d_{\varphi }\left(X_{N},Y_{N}\right)\right\}=0$.

**Fig 1:**
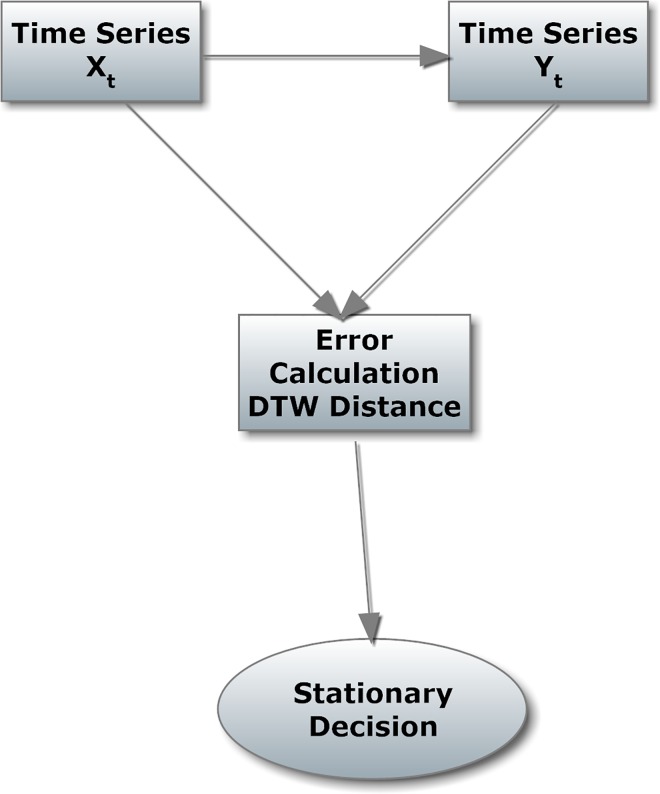
Flow diagram of the method.

Then, according to these definitions, the implementation of this method can be analyzed as follows (Fig 1):

### Comparison with the adaptive KPSS test

According to the KPSS model [8,9], the null hypothesis of the stationarity trend corresponds to the hypothesis that the variance of the random walk (r_t_) equals zero, which is expressed by the following equations.

$$y_{t}=\xi \;*\;t+r_{t}+\varepsilon_{t},$$ (9)

$$where\;r_{t}=r_{t-1}+u_{t},$$ (10)

and *t* = 1,2,3,…,*T*, which are expressed in terms of the number of observations.

Thus, if we suppose that the null hypothesis is determined to be *ξ*_*t*_ = *ξ*_0_, i.e., is constant or $H_{0}:\sigma_{e}^{2}$, this hypothesis defines the stationary process against the hypothesis, $H_{A}:\sigma_{e}^{2}>0$ which defines the nonstationary process. assuming that *e*_*t*_ are the estimated errors, which are computed as the residuals of regression {*y*_*t*_} and are given by $e_{t}=y_{t}-\bar{y}$, then the value of KPSS is calculated using Eq (11).

$$KPSS=\frac{T^{-2}\sum_{t=1}^{T}S_{t}^{2}}{{\hat{\sigma }}^{2}\left(\pi \right)},$$ (11)

where p is the order (lag) of an autoregressive AR(1) model. In other words, the partial autocorrelation at lag *p* is equal to the estimated AR coefficient in an autoregressive model with *p* coefficients [8]. The parameters of Eq 11 are defined as follows.

$$S_{t}=\sum_{j=1}^{t}e_{j},$$ (12)

where *σ*^2^ is the long-run variance of *e*_*t*_,

$$with\;\sigma^{2}=\lim T^{-1}E\left[S_{T}^{2}\right]$$ (13)

$$and\;{\hat{\sigma }}^{2}\left(p\right)=\frac{1}{T}\sum_{t=1}^{T}e_{t}^{2}+\frac{2}{T}\sum_{j=1}^{p}w_{j}\left(p\right)\sum_{t=j+1}^{T}e_{t}e_{t-j}$$ (14)

is the consistent estimator of *σ*^2^, which can be constructed from the residuals *e*_*t*_,

where *p* is the truncation lag and

$$w_{j}\left(p\right)=1-\frac{j}{\left(p+1\right)}$$ (15)

is the optional weighing function that corresponds to the specific choice of window [10].

## Experimental Results

**Fig 2:**
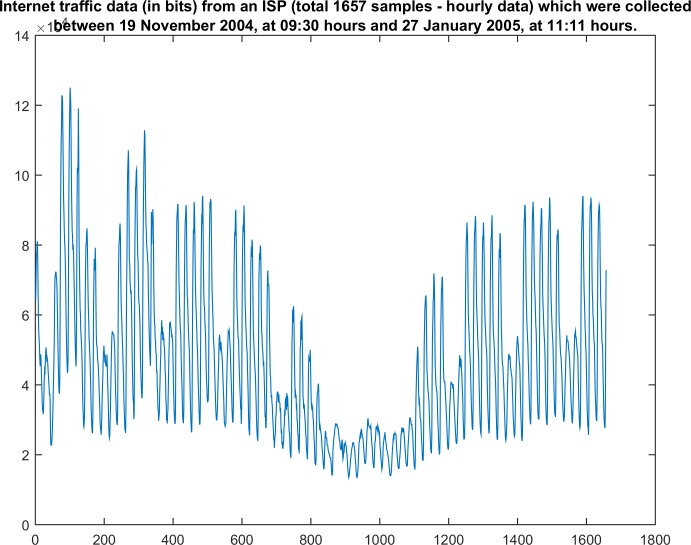
The time-series data set [17].

For the input data, a long data set *y*_*t*_ of sample size T = 1560 was selected from the Time Series Data Library [17, 27]. This data set contains internet traffic data (in bits) from an internet service provider (ISP) and aggregated traffic in the backbone of the United Kingdom academic network. It was collected between 19 November 2004 (09:30 hours) and 27 January 2005 (at 11:11 hours) as hourly data [17, 27]. This interval was chosen because this time series contains segments with (non-)stationary features (Fig 2).

**Fig 3:**
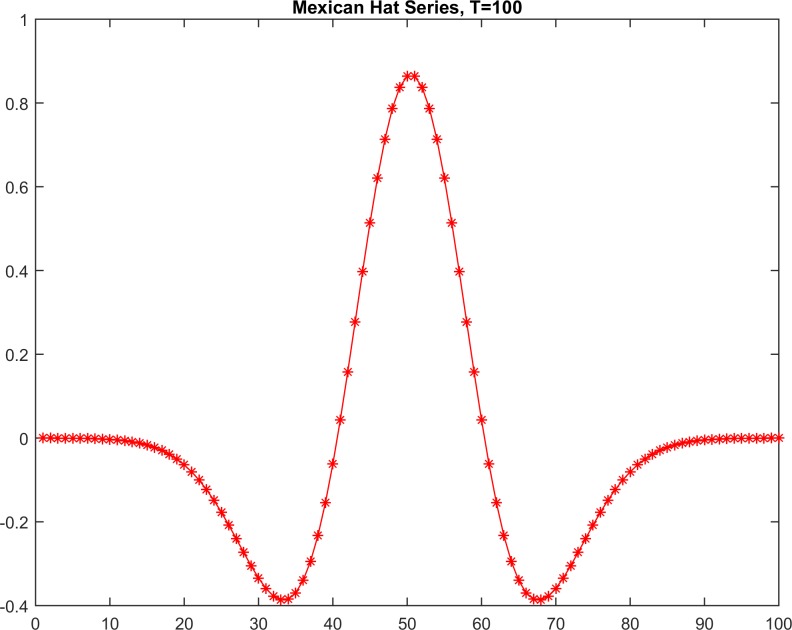
The stationary time-series data set [13,13d].

**Fig 4:**
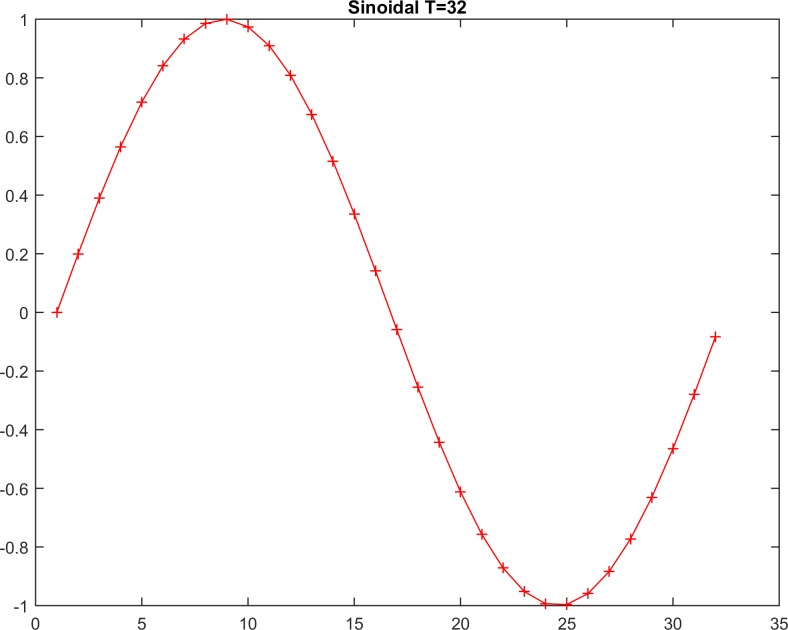
The non-stationary time-series data set.

Additionally, for calibration, we tested both methods on a classic stationary-shaped function [28,29] called the “Mexican Hat”, which was implemented in the numerical series to investigate the reliability and sensitivity of the tested methods with respect to the detection of a classic simulated stationary series (Fig 3 and Table 1). Thus, these simulated data were generated into T = [20, 40, 60, 100, 500] values to investigate the abilities of both methods to recognize this classic simulated stationary series as being stationary (Table 1). Similarly, the methods were also tested on a classic non-stationary function [28,29] called the “sinusoidal-shape” using simulated data with T = [32, 63, 126, 629, 1527] values (Fig 4 and Table 2).

**Table 1:** Results of the KPSS and RSP methods using the simulated stationary series (Mexican Hat).

id	Sample Size of Stationary Mexican Hat Data	KPSS				Dist
		Lags	hp	p-v	t-stat	
1	T = 20	0	0	0.0895	0.1246	0
		1	0	0.1000	0.0742	
		2	0	0.1000	0.0654	
		3	0	0.1000	0.0710	
		4	0	0.1000	0.0873	
		5	0	0.1000	0.1137	
		6	0	0.0485	0.1478	
		7	0	0.0220	0.1841	
		8	1	0.0100	0.2177	
2	T = 40	0	1	0.0100	0.2556	0
		1	0	0.0738	0.1332	
		2	0	0.1000	0.0950	
		3	0	0.1000	0.0783	
		4	0	0.1000	0.0706	
		5	0	0.1000	0.0679	
		6	0	0.1000	0.0686	
		7	0	0.1000	0.0721	
		8	0	0.1000	0.0781	
3	T = 60	0	1	0.0100	0.3882	0
		1	0	0.0169	0.1976	
		2	0	0.0690	0.1357	
		3	0	0.1000	0.1061	
		4	0	0.1000	0.0895	
		5	0	0.1000	0.0796	
		6	0	0.1000	0.0736	
		7	0	0.1000	0.0701	
		8	0	0.1000	0.0686	
4	T = 100	0	1	0.0100	0.6542	0
		1	1	0.0100	0.3292	
		2	1	0.0010	0.2218	
		3	0	0.0310	0.1688	
		4	0	0.0654	0.1377	
		5	0	0.1000	0.1174	
		6	0	0.1000	0.1034	
		7	0	0.1000	0.0934	
		8	0	0.1000	0.0860	
5	T = 500	0	1	0.0100	3.3201	0
		1	1	0.0100	1.6605	
		2	1	0.0100	1.1074	
		3	1	0.0100	0.8311	
		4	1	0.0100	0.6654	
		5	1	0.0100	0.5550	
		6	1	0.0100	0.4762	
		7	1	0.0100	0.4172	
		8	1	0.0100	0.3714	

**Table 2:** Results of the KPSS and RSP methods using the simulated non-stationary series (sinusoidal).

id	Sample Size of Stationary Mexican Hat Data	KPSS				Dist
		Lags	hp	p-v	t-stat	
1	T = 32	0	1	0.0100	0.3862	15.7306
		1	0	0.0122	0.2100	
		2	0	0.0455	0.1514	
		3	0	0.0918	0.1234	
		4	0	0.1000	0.1082	
		5	0	0.1000	0.0998	
		6	0	0.1000	0.0955	
		7	0	0.1000	0.0943	
		8	0	0.1000	0.0956	
2	T = 63	0	1	0.0100	0.7549	31.4446
		1	1	0.0100	0.3928	
		2	0	0.0100	0.2710	
		3	0	0.0121	0.2104	
		4	0	0.0262	0.1745	
		5	0	0.0458	0.1511	
		6	0	0.0707	0.1348	
		7	0	0.0924	0.1231	
		8	0	0.1000	0.1144	
3	T = 126	0	1	0.0100	1.5014	62.8340
		1	1	0.0100	0.7653	
		2	1	0.0100	0.5183	
		3	1	0.0100	0.3947	
		4	1	0.0100	0.3207	
		5	1	0.0100	0.2715	
		6	1	0.0100	0.2365	
		7	0	0.0121	0.2105	
		8	0	0.0196	0.1904	
4	T = 629	0	1	0.0100	7.4844	314.1597
		1	1	0.0100	3.7562	
		2	1	0.0100	2.5114	
		3	1	0.0100	1.8888	
		4	1	0.0100	1.5151	
		5	1	0.0100	1.2659	
		6	1	0.0100	1.0880	
		7	1	0.0100	0.9545	
		8	1	0.0100	0.8508	
5	T = 1257	0	1	0.0100	14.9552	628.3193
		1	1	0.0100	7.4915	
		2	1	0.0100	5.0016	
		3	1	0.0100	3.7563	
		4	1	0.0100	3.0090	
		5	1	0.0100	2.5108	
		6	1	0.0100	2.1549	
		7	1	0.0100	1.8880	
		8	1	0.0100	1.6804	

Next, the processing of these data is implemented using two processing methods: RSP and KPSS. The above-described data set [16] is segmented into data sets of various sizes ranging from 42 and 301 (Tables 3 and 4). This segmentation was performed by applying an empirical criterion. The selected weak stationary data and non-stationary segments were obtained by visually inspecting the data using the plots. Specifically, the weak stationary data were segmented using visual criteria, such as stable mean and variance. These segments have sinusoidal shapes with visually apparent random phases. This selection was adopted because these data represent ergodic and weak stationary processes [30].

**Table 3:** Results of the KPSS and RSP methods using weak-sense stationary times series.

id	Sample Size of Data with Stationary Features	KPSS				RSP Distance
		Lags	hp	p-v	t-stat	
1	T = 101 (420:520)	0	0	0.0554	0.1431	0.0085
		1	0	0.1000	0.0734	
2	T = 147 (1401:1547)	0	1	0.0100	0.3574	0.0027
		1	0	0.0226	0.1823	
3	T = 52 (61:112)	0	1	0.0100	0.3013	0.0028
		1	0	0.0426	0.1549	
4	T = 51 (260:310)	0	1	0.0100	0.3267	0.0042
		1	0	0.0293	0.1708	
5	T = 76 (452:527)	0	1	0.0100	0.2719	0.0031
		1	0	0.0596	0.1408	
6	T = 49 (572:620)	0	1	0.0100	0.2993	0.0020
		1	0	0.0409	0.1569	
7	T = 43 (608:650)	0	1	0.0100	0.2963	0.0039
		1	0	0.0404	0.1575	
8	T = 47 (693:739)	0	1	0.0100	0.3221	7.1195e-04
		1	0	0.0317	0.1679	
9	T = 109 (1411:1510)	0	1	0.0218	0.1846	0.0028
		1	0	0.1000	0.0953	
10	T = 42 (900:941)	0	1	0.0100	0.2913	3.9827e-04
		1	0	0.0441	0.1531	
11	T = 46 (900:945)	0	1	0.0100	0.3022	0.0017
		1	0	0.0403	0.1578	
12	T = 74 (1009:1082)	0	1	0.0100	0.3435	0.0039
		1	0	0.0258	0.1751	
13	T = 50 (70:119)	0	1	0.0100	0.3102	0.0032
		1	0	0.0353	0.1636	
14	T = 49 (1252:1300)	0	1	0.0100	0.3025	0.0025
		1	0	0.0398	0.1582	
15	T = 99 (1252:1350)	0	1	0.0415	0.1563	0.0026
		1	0	0.1000	0.0807	
16	T = 51 (1300:1350)	0	1	0.0100	0.3185	0.0023
		1	0	0.0333	0.1661	
17	T = 44 (1355:1398)	0	1	0.0100	0.2949	0.0011
		1	0	0.0424	0.1551	
18	T = 119 (1411:1530	0	1	0.0100	0.1307	0.0046
		1	0	0.1000	0.0674	
19	T = 73 (1438:1510)	0	1	0.0100	0.2194	0.0029
		1	0	0.1000	0.1143	
20	T = 46 (1535:1580)	0	1	0.0100	0.2838	4.7873e-04
		1	0	0.0464	0.1503	
21	T = 51 (1580:1630)	0	1	0.0100	0.3182	0.0028
		1	0	0.0334	0.1659	
22	T = 55 (1600:1654)	0	1	0.0100	0.3991	0.0065
		1	0	0.0140	0.2054	

**Table 4:** Results of the KPSS and RSP methods using times series with non-stationary features.

id	Sample Size of Data with Stationary Features	KPSS				RSP Distance
		Lags	hp	p-v	t-stat	
1	T = 87 (1:87)	0	1	0.0100	1.3119	0.0765
		1	1	0.0100	0.6664	
2	T = 101 (100:200)	0	1	0.0100	0.3685	0.1876
		1	0	0.0198	0.1900	
3	T = 71 (150:220)	0	1	0.1000	0.2375	0.0774
		1	0	0.0912	0.1237	
4	T = 101 (200:300)	0	0	0.0468	0.1499	0.1034
		1	0	0.1000	0.0769	
5	T = 101 (300:400)	0	1	0.0100	0.4739	0.0251
		1	1	0.0100	0.2411	
6	T = 51 (500:550)	0	1	0.0100	0.3422	0.0220
		1	0	0.0239	0.1790	
7	T = 31 (550:580)	0	1	0.0100	0.3375	0.2557
		1	0	0.0237	0.1794	
8	T = 51 (670:720)	0	1	0.0100	0.4438	0.0248
		1	1	0.0100	0.2363	
9	T = 101 (700:800)	0	1	0.0100	0.2662	0.0171
		1	0	0.0682	0.1362	
10	T = 51 51(800:850)	0	1	0.0100	0.2712	0.0520
		1	0	0.0600	0.1406	
11	T = 101 (1050:1150)	0	1	0.0100	0.2427	0.0359
		1	0	0.0889	0.1250	
12	T = 61 (1160:1220)	0	1	0.0100	0.3341	0.0318
		1	0	0.0286	0.1717	
13	T = 31 (1220:1250)	0	1	0.0100	0.2791	0.1863
		1	0	0.0412	0.1566	
14	T = 101 (1200:1300)	0	1	0.0100	0.2850	0.0850
		1	0	0.0496	0.1460	
15	T = 151 (1300:1450)	0	1	0.0100	0.8690	0.0172
		1	1	0.0100	0.4447	
16	T = 201 (800:1000)	0	1	0.0100	1.2795	0.0201
		1	1	0.0100	0.6561	
17	T = 101 (350:450)	0	0	0.0211	0.1863	0.0284
		1	0	0.1000	0.0962	
18	T = 101 (650:750)	0	1	0.0100	0.8997	0.0253
		1	1	0.0100	0.4679	
19	T = 81 (20:100)	0	1	0.0100	1.9414	0.1633
		1	1	0.0100	0.9843	
20	T = 200 (1:200)	0	1	0.0100	1.7559	0.0247
		1	1	0.0100	0.8890	
21	T = 301 (700:1000)	0	1	0.0100	1.1082	0.0327
		1	1	0.0100	0.5633	
22	T = 131 (170:300)	0	1	0.0100	0.5060	0.0299
		1	1	0.0100	0.2592	
23	T = 102 (1550:1650)	0	1	0.0100	0.2702	0.0138
		1	0	0.0628	0.1391	
24	T = 100 (60:160)	0	1	0.0100	0.4379	0.0154
		1	1	0.0100	0.2238	

Furthermore, this segmented time series was verified using the KPSS criterion, and the null hypothesis results of the first two lags (Tables 3 and 4) were investigated. Finally, the statistical results and the series diagrams are depicted in Table 1.

### KPSS processing

The KPSS processing was implemented via the kpsstest.m function in Matlab. Via this procedure, we investigate the possible stationary positions of the candidate segment for two specific tests—0- and 1-autocovariance lags in the Newey-West estimator of the long-run variance, each conducted at a 0.1 level of significance—using a significance level of a = 0.01. Then, we calculate the KPSS value according to Eq (8) and the exact probability value (p-value). The p-value of a statistical hypothesis test indicates the probability of obtaining a value of the test statistic that is as extreme as or more extreme than that observed by chance alone. If the null hypothesis H_**0**_ is true, then the p-value determined by a KPSS test will be low, indicating an increased probability of rejecting the hypothesis of stationarity. In this case, the null hypothesis H_**o**_ (hp = 0) is accepted when the p-value, which is calculated using the kpss.m function of Matlab, is less than or equal to 0.01. For each candidate segment, two p-values are calculated for each lag (0 and 1) (Tables 2 and 3), whereas for the Mexican Hat, nine lags (0–9) are used (Table 1) [9].

### RSP processing

**Fig 5:**
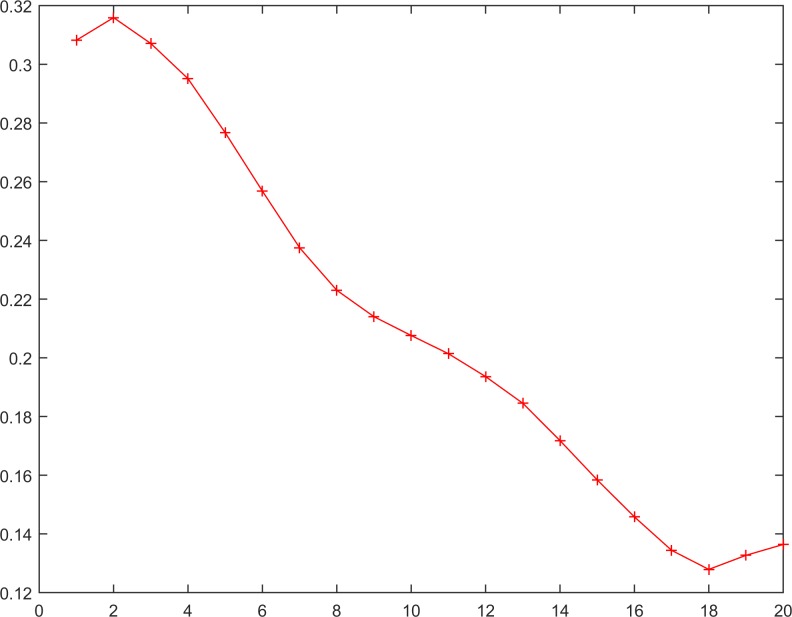
The time series X_N = 20._

**Fig 6:**
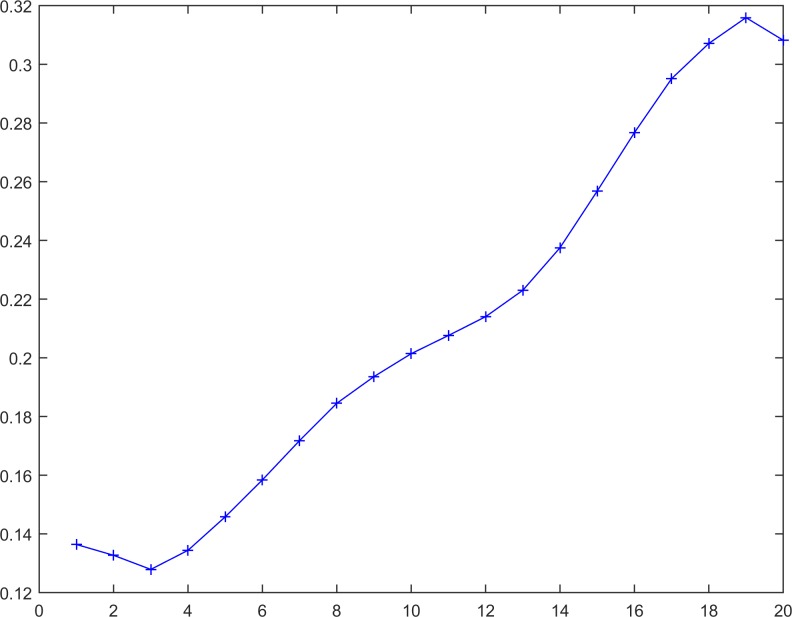
The reversible time series Y_N = 20._

The RSP processing is implemented in 3 steps according to the procedure depicted in Fig 1. Specifically, in the first step, the selected time series is processed using a reversible procedure, according to the first and second definitions (Eqs (1) and (2)). For example, for a given time series (series 14 and Fig 5), using the reversible procedure, the reverse time series can be produced (series 15 and Fig 6).

$$X_{i=1}^{N=19}=\left\{\begin{array}{l} 0.3082\;0.3159\;0.3071\;0.2951\;0.2767\;0.2568\;0.2375\;0.2223\;0.2140\;0.2076\\ 0.2014\;0.1936\;0.1845\;0.1718\;0.1584\;0.1458\;0.1344\;0.1279\;0.1327\;0.1364 \end{array}\right\}.$$ (16)

$$Y_{i=1}^{N=19}=\left\{\begin{array}{l} 0.1676\;0.1327\;0.1279\;0.1344\;0.1458\;0.1584\;0.1718\;0.1845\;0.1936\;0.2014\\ 0.2076\;0.2140\;0.2223\;0.2375\;0.2568\;0.2767\;0.2951\;0.3071\;0.3159\;0.3082 \end{array}\right\}.$$ (17)

**Fig 7:**
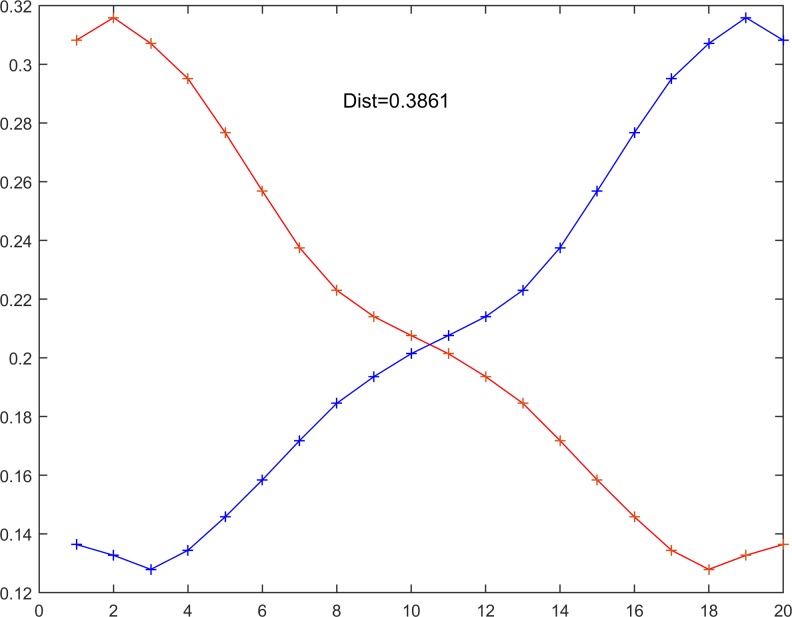
The time series X_N = 20_ and Y_N = 20_ used in the comparative DTW procedure.

In the second step, DTW = 0.3661 is calculated between the two time series using Eq (6), which is implemented via the dwt.m function of Matlab (Fig 7).

Finally, in the third step, the result of the characterization of the investigated time series X_n_ is extracted according to the evaluation procedure; this result is analyzed in Section 4.

## Results

As seen in Table 1, the decision hypothesis with H_**0**_ = 0, i.e., the acceptance decision for stationarity, depends on the sample size (Table 5) because the minimum-order lag increases dramatically according to a proportional relation. In contrast, for the RSP distance, the decision hypothesis depends on the sample size that consistently yields a zero RSP distance. Furthermore, in Tables 2 and 6, the decision hypothesis with H_**0**_ = 1, i.e., the rejection decision for stationarity, is presented for all five cases from the first lag. However, the rejection is very strong for segments with sizes exceeding 100. In contrast, in the RSP method, the calculated distances are much greater than one, indicating clear differentiation between the two investigated series types (Mexican Hat and sinusoid). Furthermore, in the sinusoid case (Table 6), the RSP distance is half of the corresponding sample size.

**Table 5:** Summary of the results in Table 1.

T (sample size)	KPSS Minimum-order Lags of H_0_ = 0 with a = 0.01	RSP Distance
20	0	0
40	1	0
60	1	0
100	3	0
500	>9	0

**Table 6:** Summary of the results in Table 2.

T (sample size)	KPSS Minimum-order Lags of H_0_ = 1 with a = 0.01	RSP Distance
32	1	15.7306
63	1	31.4446
126	1	62.8340
629	1	314.1597
1257	1	628.3193

Furthermore, the results shown in Tables 3 and 4 indicate that, in the non-stationary decision, KPSS is very sensitive to the calculation of the orders of the lags. The characterized case of the error decision H_**0**_ = 0 (i.e., sample segment n.4 in Table 3, which is referred to as segment (200:300) in the time series in the ISP database). In contrast, the RSP distance yields stationary progress <10^**−2**^, whereas the non-stationary progress is >10^**−1**^. The statistical verification of this remark is provided in Section 4.

The most obvious method of estimating the similarity or dissimilarity between time series is to calculate a metric distance directly. In this case, the DTW between the original series is selected. For a small data set, this method may be feasible. However, for large data sets, it is problematic because the time complexity is O(n*N), where n is the number of features that must be obtained for each time series, and N is the number of time series in the data set. To calculate the similarity and index efficiently, many techniques for dimensionality reduction, such as the discrete Fourier transform (DFT), the piecewise aggregate approximation (PAA), and the discrete wavelet transform (DWT), have been proposed. These techniques allow a time series X of arbitrary length n to be represented with a time series of length w, where w< n [18].

The PAA is a very simple dimensionality-reduction method for time-series mining. For the time series *X*_*N*_ (Eq (1)), a new reduction time series *X*_*M*_ is obtained, which is calculated using the following equation:

$${}_{i=1}^{\;M}|X_{i}=\frac{M}{N}\sum_{j=n/M(i-1)+1}^{\left(N/M\right)i}X_{j}.$$ (18)

This reduces the dimensionality from N to M by first dividing the original time series into M equally sized frames and then calculating the mean values for each frame. The sequence assembled from the mean values is the PAA approximation (i.e., transform) of the original time series. As shown in [31], the complexity of the PAA transform can be reduced from O(n*N) to O(n*M), where n is the number of frames.

In this case, the proposed method is tested using the PAA method to examine the efficiency of this method with respect to dimensionality reduction. Then, eight examples from Tables 3 and 4 are tested by attempting to reduce their dimensionality by ~50%, and the results are presented in Table 7. Furthermore, the results presented in Tables 1 and 2 demonstrate the efficiency of this dimensionality reduction because the stationary (Table 1) and non-stationary (Table 2) cases were tested using the same curves (Mexican hat and sinusoidal) with different sizes (20–1257) and yielded stable decisions. In Table 1 (stationary case), the reduction of the dimensionality from 500 to 20 yielded a value of zero in every case, unlike the KPSS method, which showed sensitivity to this variation. The same stable results were obtained for the results shown in Table 2 (non-stationary case), with dimensionality reduction from 1257 to 32. This case, the obtained distances are >15, and the KPSS method suffered from the same problems with the decision as mentioned for Table 1.

**Table 7:** Dimensionality reduction results obtained using the PAA algorithm for RSP methods applied to times series.

Table References	Size of Sample Data with Non-Stationary Features	RSP Distance (Original)	RSP Distance (From PAA)	Error Distance
Table 3	101(420:520)	0.0085	0.0090	0.0005
Table 3	51(260:310)	0.0042	0.0048	0.0006
Table 3	51(1300:1350)	0.0023	0.0032	0.0010
Table 3	51(1580:1630)	0.0028	0.0045	0.0017
Table 3	76(452:527	0.0031	0.0038	0.0005
Table 4	200(1:200)	0.0247	0.0242	0.0005
Table 4	101(60:160)	0.0154	0.0161	0.0007
Table 4	301(700:1000)	0.0327	0.0333	0.0006
Table 4	81(20:100)	0.1633	0.1653	0.0020
Table 4	101(1050:1150)	0.0359	0.0306	0.0053

As shown by the results in Table 7, the error of the distances between the low-dimensional data and the original time series is very small (<0.005) and, in every case, the decisions on non-stationarity or weak stationarity remain the same, as indicated in Tables 3 and 4.

### Statistical processing

In this section, the populations of the variances, means and medians of the calculated distances (Tables 3 and 4), which were extracted separately for both classes (stationary and non-stationary), are investigated (Table 7). This investigation focuses on the following two queries:

1. Why is the diversity higher than those of the two aforementioned populations?
2. What is the sample size required for the calculated diversity to achieve statistical accuracy?

Answering these queries could involve accurately measuring of the classification of the time series, i.e., stationary or non-stationary. To this end, the two-sample F-test for equal variances (homogeneity) is adopted because the hypothesis of the homogeneity of variance is known to be significant in the classification stage of discrimination inquiries. Implementing this process returns a test decision for the null hypothesis H_**0**_ according to the data in vectors x and y, which states that x and y are drawn from normal distributions with the same variance [32]. The alternative hypothesis H_**A**_ is that they come from normal distributions with different variances. The result h will be 1 if the test rejects the null hypothesis at the 5% significance level and 0 otherwise. Thus, by using this test, the possible diversity of variances for the aforementioned populations is obtained. The F-test is calculated according to Eq (19):

$$F=\frac{\sum_{i=1}^{n}{\left({\overset{⌢}{y}}_{i}-\bar{y}\right)}^{2}/k}{\sum_{j=1}^{k}\sum_{i=1}^{nj}{\left(y_{ij}-{\hat{y}}_{i}\right)}^{2}/\left(n-k-1\right)}.$$ (19)

Under the null hypothesis, the test statistic *F* has an *F*-distribution with degrees of freedom (equal to *n*_**1**_−1) in the numerator and degrees of freedom (equal to *n*_**2**_−1) in the denominator, where *n*_**1**_ and *n*_**2**_ are the sample sizes of the two data sets (x and y). Using the data in Table 4, the coefficient can be calculated via Eq (13): F = 6.4240e-04 with p = 4.4234e-26; the confidence intervals for the x and y vectors are 0.0003 and 0.0016, respectively, and DF = 19. Thus, H_**0**_ is strongly rejected because $F<F_{1-a,n_{1}-1,n_{2}-1}=2.53$ according to a two-tailed test.

Answering the second query could solve crucial issues regarding the collection of difficult-to-collect sampling data, such as medical signals. One important aspect of designing an experiment is determining how many observations are needed to draw conclusions with sufficient accuracy and confidence. The required sample size depends on many factors, including the type of experiment being contemplated, how it will be conducted, the available resources, and the desired sensitivity and confidence. Thus, the required sample sizes for the aforementioned two populations (stationary and non-stationary) are identified via an iterative procedure, resulting in a series of successively improving estimates of the required n [32]. To this end, the following equation is used:

$$n=\frac{2s_{p}^{2}t_{a(2),2(n-1)}^{2}}{d^{2}},$$ (20)

$$where\;s_{p}^{2}=\frac{SS_{1}+SS_{2}}{v1+v2}$$ (21)

and SS is the sum of the squares of the deviations from the mean. This values is called the sum of squares and is defined as $SS_{1}={\sum_{i=1}^{i=n_{1}}\left(x_{i}-\bar{x}\right)}^{2}$ and $SS_{2}={\sum_{i=1}^{i=n_{2}}\left(y_{i}-\bar{y}\right)}^{2}$ with *v*_1_ = *n*_1_−1 and *v*_2_ = *n*_2_−1.

Then, if we set *d* equal to half the width of the confidence interval [8], the interval limits for the difference between the two “populations means” can be estimated, and *d* is approximately $d=\frac{\left|\bar{x}-\bar{y}\right|}{2}$ with 2(*n*−1) degrees of freedom.

Suppose the difference between the populations is *μ*_1_−*μ*_2_. For 95% confidence, the interval must be no wider than $d=\frac{\left|0.0028-0.0730\right|}{2}=0.0351$. Therefore, *SS*_1_ = 6.1009e-05, *SS*_2_ = 0.0950, and the following quantity is determined using Eq (12):

$$s_{p}^{2}=\frac{6.1009e-05+0.950}{19+19}=0.0025.$$

Then, let us suppose that a sample size of 50 is necessary, with 2(50−1) = 98 degrees of freedom. Thus, *t*_0.05(2),98_ = 1.984. According to Eq (14), the limiting sample size n can be calculated using an iterative procedure [16]. First, we calculate

$$n=\frac{2\;*\;0.0025^{2}\;*\;1.984^{2}}{0.0351^{2}}=15.9983.$$

Next, it is possible to determine the estimate using *n* = 16, for which *t*_0.05(2),30_ = 2.042.

$$n=\frac{2\;*\;0.0025^{2}\;*\;2.042^{2}}{0.0351^{2}}=16.9473.$$

Repeating the procedure allows estimates that n = 17, for which *t*_0.05(2),32_ = 2.037.

$$n=\frac{2\;*\;0.0025^{2}\;*\;2.037^{2}}{0.0351^{2}}=16.8644.$$

According to the proposed convergence procedure [8], the value of the sequential iteration is less than 0.1. Therefore, a sample of size at least 17 (i.e., more than 16) should be obtained from each of the two populations to achieve the specified confidence interval (see Table 8).

**Table 8:** Summary of the results in Tables 3 and 4.

x (Stationary) (Table 2)	y (Non-Stationary) (Table 3)
0.0085	0.0765
0.0027	0.1876
0.0028	0.0774
0.0042	0.1034
0.0031	0.0251
0.0020	0.0220
0.0039	0.2557
7.1195e-04	0.0248
0.0028	0.0171
3.9827e-04	0.0520
0.0017	0.0359
0.0039	0.0318
0.0032	0.1863
0.0025	0.0850
0.0026	0.0172
0.0023	0.0201
0.0011	0.0284
0.0046	0.0253
0.0029	0.1633
4.7873e-04	0.0247

## Conclusion

This paper investigates the measurement of the stationarity distance of a stochastic series. The proposed method is based on the assumption that each series deviates from a stationary state and that the series would itself be stationary if it satisfied a particular condition. This particular condition is expressed by a reversible property, which contains the stationary series. The estimated deviation of this condition is called the stationarity distance or measurement error between mirror time series. This distance is based on a novel stationary ergodic process, in which the stationary series has reversible symmetric features and is calculated using the DTW algorithm via a self-correlation procedure.

Additionally, for verification, this method is compared with the KPSS test. The results of this comparison indicate that the proposed method solves several problems associated the KPSS test, including those relating to the sample size and the prediction of the order lag on which the null hypothesis is based.

Furthermore, to obtain additional statistical evidence supporting the utility of this method, as a statistical control, the F-test was used. Resolving the problem of the introduction of a sample of limited size is a topic for future research. The testing results for both methods regarding the simulated stationary series (Mexican Hat) and non-stationary (sinusoidal) series revealed the superiority of the RSP method, particularly for large sample sizes >100 (Tables 1, 2, 5 and **6**). Additionally, the test performed using real data verified the weaknesses of KPSS, particularly for some cases (e.g., cases 4 and 17; Table 4) in which the null hypothesis is accepted for a visually verified non-stationary time series. Furthermore, in both tests, the RSP showed good agreement with the expected results. Additionally, the results of the F-test showed that the RSP-distance testing groups (weak-stationary in Table 3 and non-stationary in Table 4) have non-homogeneous properties, i.e., the distances of each group are drawn from normal distributions with different variances. This finding indicates that the selected distance populations can be differentiated from each other at the 95% confidence level. Furthermore, the selected sample size of each group (20) was found to be sufficient for this statistical analysis because it is greater than 17, the value calculated in Section 4.

## Future Research

This method could be applied to additional time-series difference data to investigate the variation in the stationarity distance over time. This research could be valuable for making predictions using diagnostic medical data. For example, electroencephalography (EEG) is a noninvasive and accessible method that is widely used to measure brain function and make inferences about regional brain activity. The stationarity of EEG has been studied by many researchers, but the stationarity of EEG segments with event-related potentials (ERPs) remains concerning in many abnormal cases. Thus, the proposed method could provide a practical solution for measuring the stationarity of EEG segments very quickly and accurately.

Furthermore, this method could be used in directed weighed-complex networks. For example, in a typical study [33], the weighed-complex network is constructed using the phase-space distance calculation. This model could be modified to use RSP instead of the phase-space distance to spatially depict the stationarity properties of the investigated times series, which could have very important applications, such as EEG and seismography.
